# Complex clinical encounter series: osteoporosis presenting during pregnancy and lactation: wait and reassess

**DOI:** 10.1093/jbmr/zjae038

**Published:** 2024-03-04

**Authors:** Christopher S Kovacs

**Affiliations:** Faculty of Medicine – Endocrinology, Memorial University of Newfoundland, St. John’s, Newfoundland A1B 3V6, Canada

**Keywords:** osteoporosis, diseases and disorders of/related to bone, disorders of calcium/phosphate metabolism, nutrition, anabolics, therapeutics, antiresorptives

## Abstract

Two months after her first pregnancy, a 35-yr-old exclusively breastfeeding woman bent to move her baby in the car seat and experienced sudden, severe pain from 5 spontaneous vertebral compression fractures. Genomic screen was negative but she had mild ankylosing spondylitis previously well controlled on etanercept. She was vegetarian with a high phytate intake. A lactation consultant had advised her to pump and discard milk between feeds, leading her to believe she produced twice as much milk as her baby ingested. She presented with a LS Z score of −3.6 and a TH Z score of −1.6. After 6 mo postweaning, she was treated with teriparatide (14 mo intermittently over 18 mo) and ultimately achieved a 50% increase in LS bone density and an 8% increase in TH bone density. Her fragility is explained by normal lactational bone loss amplified by excessive milk production and phytate-induced impairment of intestinal calcium absorption, ankylosing spondylitis, and the bend-and-lift maneuver. The marked increase in bone density resulted from the combined effects of spontaneous recovery and pharmacotherapy. Spontaneous recovery of bone mass and strength should occur during 12 mo after weaning in all women, including those who have fractured.

## Case description

After an uneventful first pregnancy at age 35, this woman began exclusively breastfeeding (ie, all baby’s nutrition was from breast milk). A lactation consultant advised that due to an impression of slow weight gain in the baby, she should stimulate milk production by pumping and discarding in between feeds. At no time did the baby receive formula, nor did the pediatrician or family doctor ever express any concerns over the baby’s progress. She continued to pump-and-discard and estimated that as much milk was pumped but not used as the baby received; consequently, she produced much more milk than her baby demanded.

Two months into exclusive breastfeeding, she developed back pain that rapidly became severe and disabling. The pain was initially thought to be due to her ankylosing spondylitis, which had been well controlled on etanercept continuously since the diagnosis 2 yr earlier. Etanercept was switched to certolizumab, and a single injection of triamcinolone was given, all without relief. Then she bent over to move her baby in the car seat, and both heard and felt a loud crack in her back.

Radiographs demonstrated compression fractures of 30% at T11 and 40% at T12, accompanied by endplate depressions at L2, L3, and L4. There were bridging anterior syndesmophytes at L4-5 and L5-S1. Pre-pregnancy radiographs had shown no fractures.

She had mild asthma requiring very occasional use of inhalers, attention deficit hyperactivity disorder, postpartum depression, iron deficiency, psoriasis, prior urinary tract infections and cystitis, and periodic vertigo. She had an ulnar fracture at age 12 when she was pushed to the ground in the schoolyard, and an ankle fracture at age 17 when she fell downstairs. There was no history of an eating disorder or prolonged amenorrhea, and no family history of skeletal fragility.

She stood 159.6 cm and weighed 74.1 kg; she had lost about 2.5 cm in height. Physical exam was otherwise unremarkable. A workup for secondary causes of bone loss was unremarkable. Whole genome sequencing found no mutations in any genes related to osteoporosis, bone mass, or bone metabolism. She was HLAB27 negative.

Her calcium and vitamin D intake were estimated as adequate; however, when formally assessed several yr later by a dietitian, she was noted to be vegetarian with a very high phytate intake. Her calcium intake was about 750 mg daily prior to her first pregnancy, during which she took a 250 mg supplement to reach 1000 mg daily (below the recommended intake of 1200 mg daily). After the compression fractures, her calcium intake increased to 1600 mg daily. Her vitamin D intake was habitually 260 IU but with supplements from the start of pregnancy she had a total intake of 3800 IU daily and a 25OHD level of 97.8 nmol/L (39.1 ng/mL).

A DXA scan done at 6 mo postpartum, while still breastfeeding, showed severe bone loss at the LS (0.645 g/cm^2^; Z-score −3.6) with more modest deficits at the TH (0.736 g/cm^2^; Z-score −1.6) and femoral neck (0.542 g/cm^2^; Z-score -2.6). High resolution imaging (HR-pQCT) done at 8 mo postpartum showed very low trabecular bone volumes in the left radius and tibia.

## Clinical problem

Women occasionally fracture during late pregnancy, early postpartum, or while breastfeeding. In many reports, a cascade of 4–10 vertebral compression fractures has suddenly occurred. The optimal treatment is uncertain because substantial spontaneous improvement can be expected, osteoporosis treatments are generally not approved or indicated for premenopausal women, and there are no randomized trials comparing pharmacotherapy to spontaneous postweaning recovery.

## Differential diagnosis

**Figure 1:**
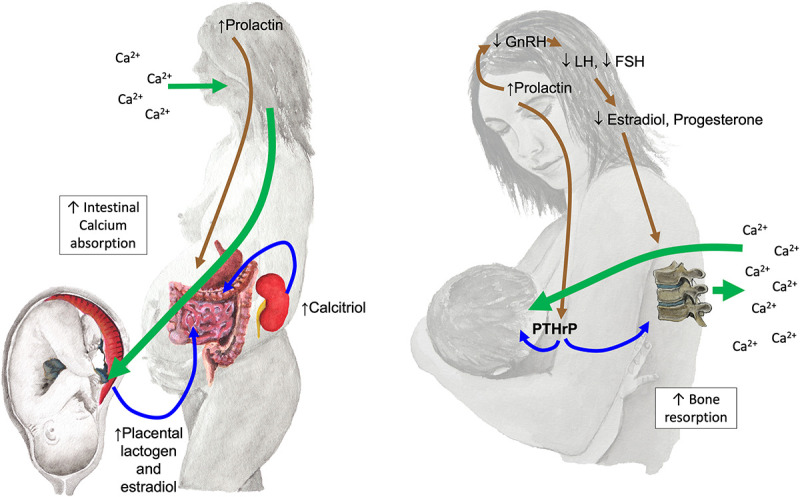
Physiological adaptations that influence bone loss during pregnancy and lactation. **During pregnancy** (left), fractional intestinal calcium absorption (long green arrow) doubles to meet the fetal calcium demand. A 2- to 5-fold increase in calcitriol (coming from the maternal kidneys) is a key driver; other factors stimulate intestinal absorption independent of calcitriol (100-fold increased estradiol from ovaries [not shown] and placenta; placental lactogen; prolactin). Normally little if any skeletal resorption occurs, but if maternal calcium intake is insufficient, then skeletal resorption must ensue to meet the fetal needs. **During lactation** (right), suckling and high prolactin suppress the hypothalamic–pituitary–ovarian axis to cause very low estradiol; these 3 factors combine to stimulate PTHrP production from the breasts, which surges in the maternal circulation and passes into milk. The combination of low estradiol and high PTHrP, and possibly other factors, stimulates bone resorption. Most of the calcium content of milk comes from the maternal skeleton (long green arrow) with the dietary calcium intake having little influence on this process. GnRH, gonadotropin-releasing hormone; LH, luteinizing hormone; FSH, follicle stimulating hormone; PTHrP, parathyroid hormone-related protein.

Calcium and bone physiology during pregnancy and lactation (Figure 1) must be considered to understand why normal reproduction confers short-term risks of fragility (reference 1 has >1000 citations of primary literature).^1^

Fetuses accrete 300–350 mg of calcium daily during the late third trimester. Normally 25% of dietary calcium is absorbed; consequently, these fetal demands exceed what non-pregnant women absorb from a 1200 mg calcium diet.^1^ However, during pregnancy, intestinal calcium absorption normally doubles to more than meet the combined needs of mother and fetus (Figure 1).^1^

Conversely, the 210 mg calcium provided to milk daily during the first 6 mo is largely resorbed from the maternal skeleton (Figure 1), independent of maternal calcium intake (confirmed by randomized trials and observational studies).^1^ The trabecular-rich LS typically loses 5%–10% of aBMD over 6 mo of near-exclusive lactation with half that amount lost from the TH.^1^ After weaning, aBMD of the maternal skeleton is usually fully restored over the succeeding 12 mo.^1^

During pregnancy, insufficient calcium intake or absorption must induce skeletal resorption to meet the fetal requirements.^1^ During lactation, the daily volume of milk produced is the most significant determinant of the amount of bone resorbed. Consequently, greater milk output causes greater bone loss.^1^

The foregoing explains why transient skeletal fragility can occur during pregnancy and lactation. Fractures are also precipitated by increased mechanical strain from the pregnancy-related weight gain, exaggerated lumbar lordosis, and the carrying and bending maneuvers with the baby and associated paraphernalia (eg, car seat, stroller). One compression fracture creates instability that can precipitate a cascade of fractures.^2^

Table 1 lists causes of fragility that have been identified in women who fractured during pregnancy or lactation. The normal physiological demands of pregnancy and lactation may unmask underlying skeletal fragility. In some cases, PTHrP release has been excessive and caused hypercalcemia with increased bone loss.^1^

**Table 1:** Differential diagnosis of skeletal fragility during pregnancy and lactation.

**A. PREGNANCY**
With systemic skeletal resorption and/or fragility
Mechanical issues
(eg, petite frame, low body weight, low peak bone mass, lordotic posture, increased weight-bearing, bedrest)
Nutritional
(eg, low calcium intake; low vitamin D; high phytate intake; anorexia)
Excess PTHrP from placenta and/or breasts during pregnancy
Gastrointestinal
(eg, celiac disease, Crohn’s disease, other malabsorptive disorders
Other hormonal causes of bone loss
(eg, primary hyperparathyroidism, hyperthyroidism, Cushing’s syndrome, etc.)
Renal
(eg, hypercalciuria/renal calcium leak; Chronic renal insufficiency)
Pharmacological
(eg, GnRH analogs, Progestin-only contraceptives, Glucocorticoids, etc.)
Rheumatic and connective tissue disorders
(eg, ankylosing spondylitis, rheumatoid arthritis, Ehlers–Danlos syndrome, etc.)
Primary disorders of bone quality
(eg, osteogenesis imperfecta, osteopetrosis, *LRP5/6* inactivating mutations, *WNT1* mutations)
2. With localized fragility and lack of systemic bone resorption
Transient osteoporosis of the hip in pregnancy
**B. LACTATION**
Normal or excess lactational bone loss, mediated by PTHrP and low estradiol
Increased milk production
Mechanical (carrying, bending, and lifting maneuvers with baby and related paraphernalia)
All conditions in section A that cause bone loss or fragility during pregnancy

A separate condition to briefly consider is transient osteoporosis of the hip in pregnancy (migratory osteoporosis or bone marrow edema syndrome).^1^ It occurs in men and women with equal frequency and is not due to systemic skeletal resorption. Hip pain, limp, or fracture may occur in one or both hips in the third trimester or early postpartum. Whether this occurs coincidentally or pathophysiologically linked to pregnancy is unclear. DXA shows very low aBMD in the hip, while the LS is normal or modestly reduced (the opposite of the main subject of this review). MRI shows edema of the femoral head and marrow. The DXA and MRI findings typically resolve within 2 to 12 mo.

## Investigations in this patient (but recommended for all who fracture)

When women fracture during pregnancy or lactation, a careful history is needed. This yielded the instruction that caused an iatrogenic excess in milk output. A dietitian provided a reliable estimate of calcium intake and noted her high phytate intake.

Spine radiographs are needed to document the number and severity of vertebral compression fractures; MRI may identify fractures not seen on plain radiographs. DXA should be done as soon as possible to establish a baseline value; typically aBMD is low in the spine, while the hip may be unremarkable. There is usually no pre-pregnancy DXA scan.

A workup should be undertaken to rule out categories of conditions listed in Table 1. A more extensive workup is justifiable given the younger ages of these patients and especially with multiple and severe compression fractures. For example, a small bowel biopsy might be done to rule out malabsorptive disorders. Whole genomic screening has yielded a high prevalence of pathogenic mutations in skeletal genes such as *COL1A1, LRP5/6*, and *WNT.*^1^^,^^3^ For patients with no financial coverage, some pharmaceutical companies offer complimentary screening with 300-gene panels to patients who may have genetic skeletal disorders.

## Diagnosis

Pregnancy and lactation-associated osteoporosis with multi-level vertebral compression fractures. Excessive bone loss occurred during pregnancy due to inadequate calcium absorption (caused by high phytate intake), and during lactation due to an iatrogenic increase in milk output. A bend-and-lift maneuver precipitated a cascade of fractures. Ankylosing spondylitis can increase the risk of compression fractures,^4^ but it is less likely to be a major factor given the limited nature of her condition. Glucocorticoid-induced loss is unlikely, given the single dose administered.

## Treatment and progression

This woman was initially treated conservatively, continued to exclusively breastfeed, and gradually weaned the baby by 9 mo. Her back pain was unrelenting. An HR-pQCT scan 7 mo after the baseline (15 mo postpartum and 6 mo postweaning) showed no improvement. However, HR-pQCT threshold parameters are set to detect only fully mineralized bone. A DXA scan was not repeated at this time and so the degree to which aBMD had spontaneously recovered was not determined. The unchanged HR-pQCT parameters prompted a decision to start teriparatide. She ultimately received 14 mo of treatment over 18 mo, with stops and starts due to exacerbations of bone pain.

A repeat DXA scan at 13 mo into treatment (19 mo postweaning) showed a 50% improvement in the LS; all values were within the expected ranges for age (Supplementary Table 1).

After teriparatide, she moved to another province and sought advice about a second pregnancy. She had maintained calcium and vitamin D supplements; her ankylosing spondylitis was well controlled.

It was at this visit that the pump-and-discard advice was revealed, her very high phytate intake was discovered, and her calcium intakes were quantified. Modest repeat screening showed no other evidence of secondary causes of osteoporosis; P1NP and C-telopeptide were normal.

Repeat DXA about a year after teriparatide ended showed all values in the normal ranges for age but could not be directly compared to prior measurements (Supplementary Table 1). It appeared likely that no major changes had occurred within a year after teriparatide was discontinued. Radiographs showed the previously documented and unchanged vertebral fractures.

## Discussion

Although the skeleton is normally resorbed during lactation, this usually has no long-term consequences. More than 5 dozen epidemiological studies have found that parity and lactation are not risk factors for osteoporosis, fragility fractures, or low aBMD, but may even protect against these conditions.^1^ This is because aBMD is normally restored after weaning. The factors that regulate this recovery remain to be elucidated.^1^

However, in the short-term, some women develop fragility fractures, and usually as primigravidas.^5-7^ Approximately 70%–90% of such fractures occur while breastfeeding,^1^^,^^7^^,^^8^ reflecting that most of the bone loss occurs during lactation. Compression fractures are likely underrecognized because back pain is common during pregnancy and breastfeeding. An online survey of affected women confirmed a mean delay of 12 wk from onset of pain to a diagnostic spine radiograph being done.^8^ Published reports have largely described women with cascades of fractures but this likely represent the bias of reporting the worst cases. Most case series have only a few cases, owing to the rarity of this condition.

Among 837 347 pregnancies in Japan, 379 women presented to hospital within 2 yr of pregnancy due to fractures, for an incidence of 4.5/10 000 or 0.045%.^6^ Vertebral compression fractures occurred in 57 women (0.7/10 000 or 0.0068%). Among 1260 thoracic, lumbar, or thoracolumbar MRIs done in Turkish women of reproductive age, 6 women (0.47%) had compression fractures associated with recent pregnancy or lactation, with a mean of 5.6 fractures per woman.^5^ Other studies suggest that ankle and lower tibia fractures may be more common than vertebral fractures during pregnancy and lactation.^6^^,^^8^

In follow-up of women with recent pregnancy or lactation-associated compression fractures, aBMD spontaneously increased 10%–70%.^1^^,^^7^^,^^9^ Cessation or avoidance of breastfeeding should be encouraged to prevent further skeletal losses and allow the natural postweaning recovery to be initiated. However, weaning the baby remains the woman’s personal decision, and many have continued to breastfeed after fracturing (as did this patient).

There is an understandable urge to intervene immediately. Multiple case reports and series have described anecdotal and uncontrolled use of bisphosphonates, teriparatide, denosumab, romosozumab, strontium ranelate, calcitonin, calcitriol, and fluoride.^1^ Vertebroplasty and kyphoplasty have also been used. Increases of 10-30% in aBMD have been achieved with pharmacotherapy and assumed to be due to the treatment, but whether this exceeds what would have occurred naturally after weaning remains unknown.

Dominating the literature is the largest case series of 107 women with pregnancy and lactation-associated fractures.^7^ Of these, 81 (76%) were treated with bisphosphonates, teriparatide, or a combination of the two. Twice as many fractures subsequently occurred in those who received pharmacotherapy compared to those who did not. This might indicate adverse effects of pharmacotherapy or confounding by indication (ie, that more severely affected women received pharmacotherapy). Unfortunately, the original figure showing change in aBMD was found to be erroneously replicated from another paper.^10^ The replacement figure does not show any differences in aBMD increases of treated vs untreated women.

Overall, it remains uncertain whether pharmacotherapy is superior to conservative management, given the lack of controlled comparisons.

I recommend that affected women should be treated conservatively at first and avoid another pregnancy until the skeleton has sufficiently recovered. This comprises optimized nutrition (including calcium 1200 mg daily and vitamin D intake to achieve a 25OHD level > 75 nmol/L); correction of any malabsorptive disorders; early mobilization; weight-bearing activity; avoiding activities with significant lifting or risk of falls; considering not breastfeeding (with pregnancy fractures) or weaning the baby (with lactational fractures); physiotherapy to maintain mobility, improve core muscle strength, and reduce pain; and a temporary supportive corset for vertebral fracture pain. The extent of spontaneous recovery of LS aBMD should be assessed at 12–18 mo. HR-pQCT will be less reliable unless the parameters are adjusted to detect undermineralized bone and osteoid.

What is sufficient recovery or when and in whom should pharmacotherapy be initiated? It is difficult to advise in the absence of data. I recommend waiting for the follow-up measurement at 12–18 mo, and that pharmacotherapy might be initiated if the Z score is still below expected for age, or if there is bone pain that might be ameliorated. Pharmacotherapy may be initiated earlier if the situation is considered dire with severe bone loss and/or pain, or new or worsening fractures. I also advise that a woman who fractured should wait 12–18 mo before considering another pregnancy, to optimize skeletal strength before challenging it again with another reproductive cycle.

Why avoid immediate use of pharmacotherapy? It seems prudent to first allow the spontaneous aBMD increase to occur. Use of anti-resorptive medications could conceivably blunt postweaning recovery, given that combination therapy blunts the effect of anabolic treatment in women with osteoporosis. The interval of bone loss from lactation is over; why treat with anti-resorptives that stop bone loss? With respect to anabolics, in postmenopausal women any gains in aBMD achieved are lost in 12–18 mo unless antiresorptive treatment is started. Conversely, anecdotal data suggest that bone gains from anabolic treatment may persist in reproductive-age women without anti-resorptive treatment; however, the follow-up has been brief, as in this case. Fracture risk should otherwise be low and bone mass stable in reproductive-age women, even despite having had fractures. Use of osteoporosis medications is generally off-label in reproductive-age women, with concerns about potential teratogenic effects.

These uncertainties are why I urge that pharmacological treatment should be used after assessing the magnitude of spontaneous recovery of aBMD at 12–18 mo postweaning, or earlier in the most severe and dire cases. There are no data to suggest that interim bone turnover marker measurements would predict the magnitude of recovery. If pharmacotherapy is to be used, anabolic treatment makes more physiological sense than an antiresorptive medication.

Women can be reassured that fractures may not recur during subsequent pregnancies, especially because many documented cases have been nutritional in origin. However, recurrences have been documented in 20%–25% of cases, especially with genetic causes of skeletal fragility or where nutritional deficiencies were not corrected.

## Unanswered questions

When is pharmacotherapy needed? Should it be anabolic or antiresorptive? Is an antiresorptive needed after anabolic treatment?

## Conclusions and future directions

Many cases of osteoporosis associated with pregnancy and lactation are due to excess bone loss during lactation. Less often, excess bone resorption occurs during pregnancy from inadequate calcium intake or net calcium absorption. The mechanical and physiological demands of pregnancy and lactation can unmask disorders causing skeletal fragility. Given the absence of controlled trials, and that spontaneous recovery should occur, a wait-and-see approach seems prudent with a DXA done 12–18 mo postweaning (postpartum in women who do not breastfeed). If the aBMD response is judged to be inadequate by a Z score below expected values, or new/worsening fractures occur during postweaning, then pharmacotherapy may be indicated. An anabolic makes physiological sense in this setting but its use remains to be clarified through adequate study. A global registry of cases with verified data may prove useful.

**Key Points**

- In all pregnant women, inadequate calcium intake or absorption will cause bone loss.
- In all breastfeeding women, skeletal resorption provides much of the calcium content of milk.
- Women occasionally suffer fractures during lactation, and less often during pregnancy or early postpartum.
- Spontaneous recovery of bone mass and strength should occur during 12 mo after weaning both in women with uneventful lactation and also in women who fractured.
- Potential benefits of pharmacotherapy (when, which, in whom?) in women who fractured remain to be determined.

## Author contributions

Christopher S. Kovacs (Conceptualization, Data curation, Formal analysis, Funding acquisition, Investigation, Methodology, Project administration, Resources, Software, Supervision, Validation, Visualization, Writing—original draft, Writing—review & editing)

## Supplementary Material

SUPPLEMENTARY_TABLE_1_zjae038

## References

[1] Kovacs CS. Maternal mineral and bone metabolism during pregnancy, lactation, and post-weaning recovery. Physiol Re*v*. 2016;96(2):449–547. 26887676 10.1152/physrev.00027.2015

[2] Broy SB. The vertebral fracture Cascade: etiology and clinical implications. J Clin Densito*m*. 2016;19(1):29–34. 26363627 10.1016/j.jocd.2015.08.007

[3] Butscheidt S, Delsmann A, Rolvien T, et al. Mutational analysis uncovers monogenic bone disorders in women with pregnancy-associated osteoporosis: three novel mutations in LRP5, COL1A1, and COL1A2. Osteoporos In*t*. 2018;29(7):1643–1651. 29594386 10.1007/s00198-018-4499-4

[4] Ghasemi-Rad M, Attaya H, Lesha E, et al. Ankylosing spondylitis: a state of the art factual backbone. World J Radio*l*. 2015;7(9):236–252. 26435775 10.4329/wjr.v7.i9.236 PMC4585948

[5] Yildiz AE, Ozbalci AB, Ergen FB, Aydingoz U. Pregnancy- and lactation-associated vertebral compression fractures: MRI prevalence and characteristics. Osteoporos In*t*. 2021;32(5):981–989. 33236194 10.1007/s00198-020-05754-w

[6] Toba M, Terauchi M, Moriwaki M, Obayashi S, Miyasaka N, Fushimi K. Fractures within 2 years of an obstetric hospitalization: analysis of nationwide administrative data in Japan. J Bone Miner Meta*b*. 2022;40(5):748–754. 35690967 10.1007/s00774-022-01336-4

[7] Kyvernitakis I, Reuter TC, Hellmeyer L, Hars O, Hadji P. Subsequent fracture risk of women with pregnancy and lactation-associated osteoporosis after a median of 6 years of follow-up. Osteoporos In*t*. 2018;29(1):135–142. 28965212 10.1007/s00198-017-4239-1

[8] KondapalliAV, Kamanda-KossehM, WilliamsJM, et al. Clinical characteristics of pregnancy and lactation associated osteoporosis: an online survey study. Osteoporos In*t*. 2023;34(8):1477–1489.37204454 10.1007/s00198-023-06793-9

[9] Phillips AJ, Ostlere SJ, Smith R. Pregnancy-associated osteoporosis: does the skeleton recover? Osteoporos In*t*. 2000;11(5):449–454. 10912848 10.1007/s001980070113

[10] Kovacs CS. Fraudulent, duplicate publication of pregnancy/lactation data in Osteoporosis International. Osteoporos In*t*. 2023;34(12):2141–2142. 37855887 10.1007/s00198-023-06939-9

