# Supplementary material for: Complex clinical encounter series: osteoporosis presenting during pregnancy and lactation: wait and reassess
Source: J Bone Miner Res. 2024 Mar 4;39(3):197–201. doi: 10.1093/jbmr/zjae038 (PMC11207916; doi:10.1093/jbmr/zjae038)
Supplement: SUPPLEMENTARY_TABLE_1_zjae038 [file SUPPLEMENTARY_TABLE_1_zjae038.docx]

**SUPPLEMENTARY TABLE 1**

Areal bone density measurements. The timing in relation to post-partum, post-weaning, and teriparatide treatment is indicated. The first two measurements were done on the same GE/Lunar device. The third was done on a different GE/Lunar device and so the readings are not directly comparable. The percentage increases between the first and second measurements are shown.

|  | 6 months post-partum | 28 months post-partum  19 months post-weaning  13 months into teriparatide | 45 months post-partum  36 months post-weaning  12 months post teriparatide |
| --- | --- | --- | --- |
| L1-4 | 0.645 g/cm^2^ | 0.968 g/cm^2^ (50% increase) | 1.139 g/cm² |
|  | Z-score -3.6 | Z-score -0.6 | Z-score -0.3 |
| Total Hip | 0.736 g/cm^2^ | 0.797 g/cm^2^ (8% increase) | 0.790 g/cm² |
|  | Z-score -1.6 | Z-score -1.1 | Z-score -1.7 |
| Femoral Neck | 0.542 g/cm^2^ | 0.612 g/cm^2^ (13% increase) | 0.765 g/cm² |
|  | Z-score -2.6 | Z-score -1.9 | Z-score -1.6 |
